# Effects of senescence and angiotensin II on expression and processing of amyloid precursor protein in human cerebral microvascular endothelial cells

**DOI:** 10.18632/aging.101362

**Published:** 2018-01-15

**Authors:** Ruohan Sun, Tongrong He, Yujun Pan, Zvonimir S. Katusic

**Affiliations:** 1 Department of Neurology, the First Affiliated Hospital of Harbin Medical University, Harbin, Heilongjiang Province, 150001, China; 2 Department of Anesthesiology and Molecular Pharmacology and Experimental Therapeutics, Mayo Clinic College of Medicine, Rochester, MN55905, USA

**Keywords:** APP processing, endothelium, senescence, Ang II, BACE1 inhibitor IV

## Abstract

The present study was designed to determine the effects of senescence and angiotensin II (Ang II) on expression and processing of amyloid precursor protein (APP) in human brain microvascular endothelial cells (BMECs). Senescence caused a decrease in APP expression thereby resulting in reduced secretion of soluble APPα (sAPPα). In contrast, β-site APP cleaving enzyme (BACE1) expression and production of amyloid β (Aβ)40 were increased in senescent endothelium. Importantly, in senescent human BMECs, treatment with BACE1 inhibitor IV inhibited Aβ generation and increased sAPPα production by enhancing a disintegrin and metalloprotease (ADAM)10 expression. Furthermore, Ang II impaired expression of ADAM10 and significantly reduced generation of sAPPα in senescent human BMECs. This inhibitory effect of Ang II was prevented by treatment with BACE1 inhibitor IV. Our results suggest that impairment of α-processing and shift to amyloidogenic pathway of APP contribute to endothelial dysfunction induced by senescence. Loss of sAPPα in senescent cells treated with Ang II exacerbates detrimental effects of senescence on APP processing. Notably, inhibition of BACE1 has beneficial effects on senescence induced endothelial dysfunction. Reported findings may help to explain contributions of senescent cerebral microvascular endothelium to development of cerebral amyloid angiopathy and Alzheimer’s disease (AD) pathology.

## Introduction

Epidemiological, experimental and clinical studies have suggested that age-related cerebrovascular dysfunction plays a critical role in the pathogenesis of dementia, including Alzheimer’s disease (AD) [1–4]. The amyloid cascade hypothesis remains the most frequently invoked hypothesis to explain the pathogenesis of AD [5]. Amyloid β (Aβ), the main constituent of amyloid plaques and a key pathogenic factor in AD, has detrimental effects on cerebral blood vessels resulting in disruption of homeostatic function of the cerebrovascular endothelial cells [6–8].

Cellular senescence is an important contributor to aging and age-related diseases [9–12]. Prior studies provided evidence that processing of endogenous amyloid precursor protein (APP) is down-regulated in senescent human fibroblasts [13], but the effects of senescence on APP expression and processing in vascular endothelium have not been studied. APP is highly expressed in endothelium and can be processed by two major proteolytic pathways [14]. In the non-amyloidogenic pathway, APP is cleaved by α-secretase within the Aβ sequence thereby generating soluble APPα (sAPPα), a well-known anticoagulant, neurotrophic, and neuroprotective molecule [14–16]. In contrast, amyloidogenic processing of APP sequentially driven by β-site APP cleaving enzyme (BACE1) and γ-secretase generates cytotoxic Aβ [14]. Under physiological conditions, endothelial APP is primarily processed via non-amyloidogenic pathway [14,17]. A disintegrin and metalloprotease (ADAM)10 has been identified as the major α-secretase responsible for processing of APP [18]. Our previous studies demonstrated that in human brain microvascular endothelial cells (BMECs), ADAM10 is stimulated by activation of prostacyclin (PGI_2_)/cyclic adenosine monophosphate (cAMP) signaling pathway [16]. On the other hand, we also have demonstrated that the expression and activity of BACE1 in cerebrovascular endothelium is suppressed by endothelial nitric oxide synthase (eNOS)/nitric oxide (NO)/cyclic guanosine monophosphate (cGMP) signaling [19]. The inhibition of β-secretase by NO and stimulation of α-secretase by PGI_2_ contribute to predominant cleavage of APP via α-secretase pathway in endothelium. Under pathological conditions, β-processing of APP is activated therefore increasing production of Aβ [14,17]. Importantly, inhibition of BACE1 could prevent or reduce the accumulation of Aβ in the brain, thereby reducing AD-related pathology [20–22]. Not surprisingly, inhibitors of BACE1 are currently being developed for the treatment of AD [21,23,24]. However, the effects of BACE1 inhibitors on human brain microvascular endothelium have not been defined.

Hypertension is one of the vascular risk factors implicated in the pathogenesis of AD. Indeed, midlife hypertension increases the risk for development of AD later in life [25,26]. Multiple lines of experimental evidence suggest that hypertension-induced cerebral microvascular impairment precedes cognitive decline [2,27,28] and AD neuropathology [29–32]. However, the molecular mechanisms responsible for hypertension-induced alterations in vascular function contributing to the development of AD are poorly characterized. Angiotensin II (Ang II) is a candidate for the hypothesized mechanistic link between hypertension and AD [33]. Prior studies established that vasoconstrictor and pro-oxidant effects of Ang II contribute to pathogenesis of essential hypertension [34,35]. Furthermore, increased circulating levels of Ang II accelerate development of AD pathology by promoting β-secretase activity [32,36]. Existing literature supports the concept that Ang II increases superoxide production by activation of angiotensin II type 1 receptor (AT_1_R) in cerebral microvascular endothelium thereby causing endothelial dysfunction [37–40]. However, no previous study has assessed the direct effects of Ang II on metabolism of APP in endothelial cells of human brain blood vessels. Therefore, in this study, we determined the effects of Ang II on APP expression and its metabolism in young and senescent human brain microvascular endothelium.

## RESULTS

### Levels of APP expression and processing of APP in human BMECs during cellular senescence

**Figure 1:**
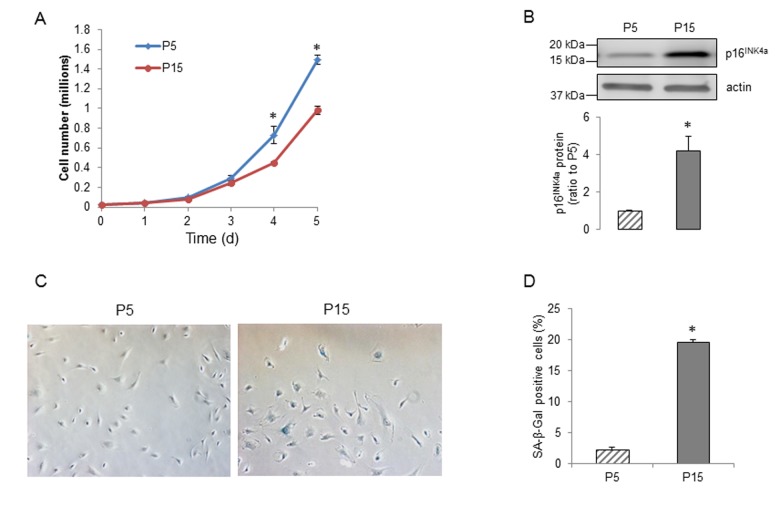
**Senescence of human BMECs**. (**A**) Growth curves of P5 and P15 human BMECs, n=3, *P<0.05, compared to P5 on the same day. (**B**) Expression of senescence-associated marker p16^INK4a^ in human BMECs, n=9. (**C**) Staining of young and senescent human BMECs for SA-β-Gal (left) and the percentage of β-gal positive cells (right), n=6. Data are presented as mean±SEM, *P<0.05, compared to P5.

**Figure 2:**
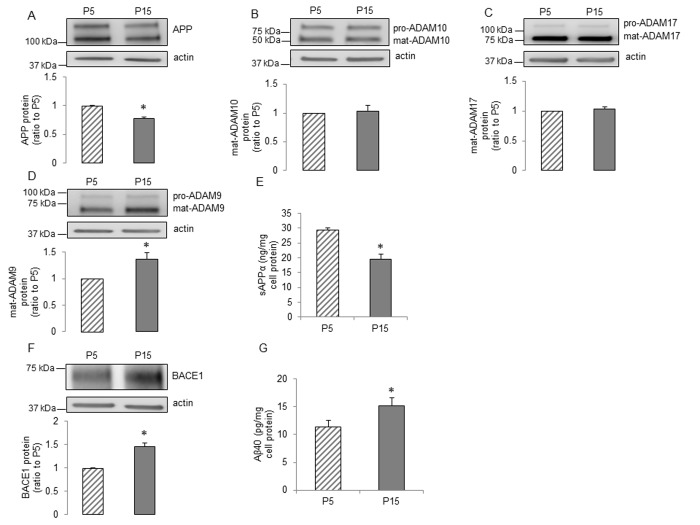
**The influence of senescence on expression and processing of APP in human BMECs.** Human BMECs were passaged 5 and 15 times. Cell lysates were subjected to Western blot. Protein levels of (**A**) APP, (**B**) ADAM10, (**C**) ADAM17, (**D**) ADAM9 and (**F**) BACE1 were measured, n=6-9. Cells were incubated in 1.5ml EGM2 for 24h. Conditioned media were collected for measuring sAPPα (**E**, n=13) and Aβ40 (**G**, n=15) via commercially available ELISA kits respectively. Data are presented as mean±SEM, *P<0.05, compared to P5.

Human BMECs were sequentially passaged and the senescent cells showed substantially slower growth rates, increasing expression of the senescence marker p16^INK4a^ and more cells positive for senescence-associated β-galactosidase (SA-β-Gal) activity (Figure 1A-D). To determine the effects of senescence on expression of APP and its metabolism in human BMECs, we examined the protein levels of APP, α- and β-secretases. APP protein levels were significantly reduced in the senescent human BMECs (Figure 2A). We assessed the expression of ADAM10, ADAM9 and ADAM17, since these three metalloproteases of the ADAM family have been suggested to exert α-secretase activity in metabolism of APP [41]. ADAM10 (but not ADAM9 or ADAM17) appears to be a major contributor to both constitutive and inducible α-secretase activity in endothelial cells [14,16,42]. We found that the expression of ADAM10 and ADAM17 were not changed (Figure 2B-C), however, senescence increased the expression of ADAM9 in passage 15 (P15) human BMECs (Figure 2D). Although there was no decrease in α-secretase expression, the secretion of sAPPα was surprisingly lower in senescent human BMECs (Figure 2E).

We next investigated senescence-associated alterations in amyloidogenic processing of APP. Western blot analysis detected a significant up-regulation of BACE1 (Figure 2F). Consistent with increased BACE1, levels of Aβ40 were significantly increased in senescent human BMECs (Figure 2G). The production of Aβ42 was undetectable (n=13, data not shown). Schematic summary of effects of senescence on expression and processing of APP is shown in Figure 6A.

### Effects of β-secretase inhibitor IV (BACE1 inhibitor IV) on APP expression and processing in young and senescent human BMECs

**Figure 3:**
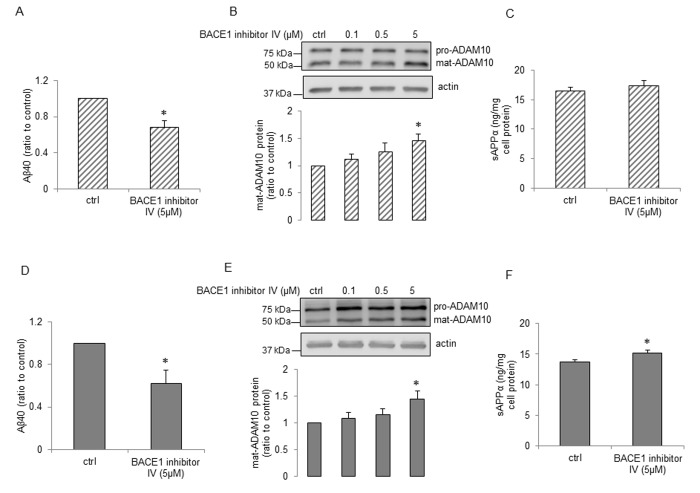
**The effects of BACE1 inhibitor IV on young and senescent human BMECs.** P5 cells (**A** and **C**) and P15 cells (**D** and **F**) were treated with 2ml EGM2 + BACE1 inhibitor IV (5μM) or 2ml EGM2 alone (ctrl) for 24h. Aβ40 (**A** and **D**, n=6-7) and sAPPα (**C** and **F**, n=6) levels from cell supernatants were analyzed using commercially available ELISA kits respectively. P5 cells (**B**) and P15 cells (**E**) were treated with BACE1 inhibitor IV with indicated concentrations for 24h. Protein samples were subjected to Western blot, n=4. Data are presented as mean±SEM, *P<0.05, compared to control.

We next determined the effects of BACE1 inhibitor IV on APP expression and its metabolism. Treatment with BACE1 inhibitor IV resulted in decrease of Aβ40 production both in young and senescent human BMECs (Figure 3A and Figure 3D, respectively). Surprisingly, BACE1 inhibitor IV caused significant increase in expression of ADAM10 (Figure 3B and Figure 3E). In senescent cells treated with BACE1 inhibitor IV, sAPPα levels were significantly up-regulated (Figure 3F). BACE1 inhibitor IV did not affect sAPPα production in young cells (Figure 3C). We further studied expressions of APP, ADAM9 and ADAM17 but no alterations of these protein levels were observed in cells treated with BACE1 inhibitor IV (SSupplemental Figure 1A-F). Schematic summary of APP expression and processing in human senescent BMECs treated with BACE1 inhibitor IV is shown in Figure 6B.

### Effects of Ang II on APP expression and processing in young and senescent human BMECs

**Figure 4:**
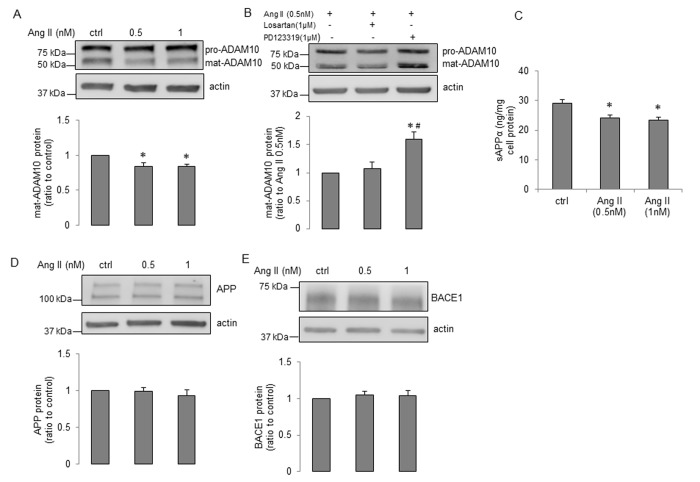
**Ang II impairs α-processing of APP in senescent human BMECs.** Cells were treated with Ang II with indicated concentrations for 24h. Cell lysates were collected for Western blot. Protein expression of (**A**) ADAM10, (**D**) APP and (**E**) BACE1 were measured, n=6-7. (**C**) Cell supernatants (1.5ml) were collected for sAPPα detection via a commercially available ELISA kit, n=11. *P<0.05, compared to control. (**B**) Senescent cells were treated with losartan (1μM) or PD123319 (1μM) for 1h, and then incubated with Ang II (0.5nM) for 24h. ADAM10 expression was measured, n=10. *P<0.05, compared to Ang II (0.5nM), ^#^P<0.05, compared to Ang II (0.5nM) plus losartan (1μM). Data are presented as mean±SEM.

**Figure 5:**
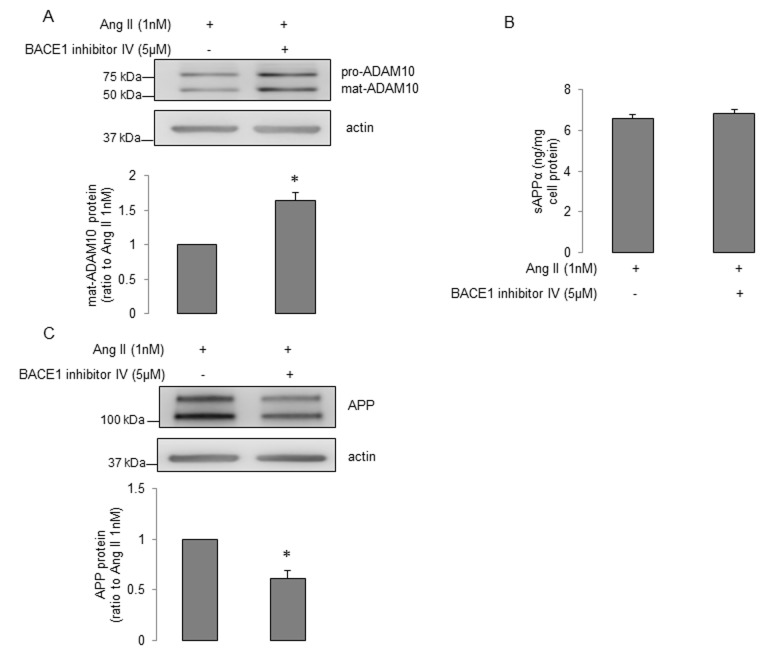
**The effects of BACE1 inhibitor IV on α-processing of APP in senescent human BMECs treated with Ang II.** Senescent BMECs were treated with Ang II (1nM) alone or Ang II (1nM) plus BACE1 inhibitor IV (5μM) for 24h. Protein samples were subject to Western blot. Protein levels of (**A**) ADAM10 and (**C**) APP were detected, n=6-7. (**B**) Conditioned media (5ml) were collected for measuring sAPPα via the ELISA kit, n=8. Data are presented as mean±SEM, *P<0.05, compared to Ang II (1nM) alone.

**Figure 6:**
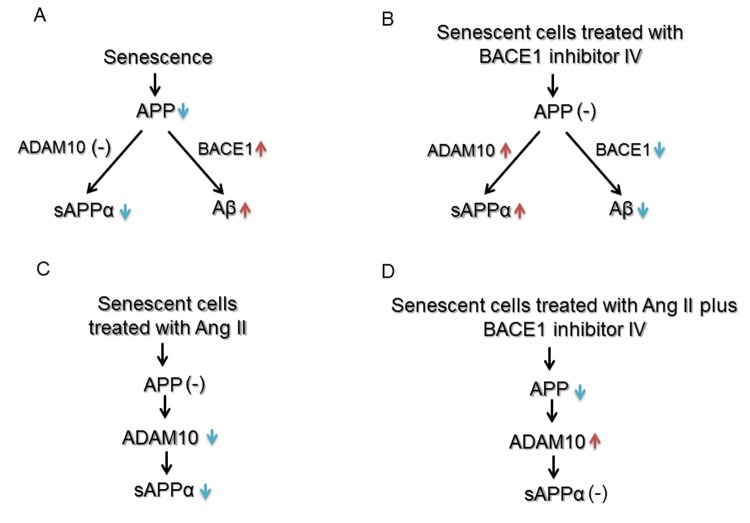
**Schematic summary of effects of senescence, Ang II and BACE1 inhibitor IV on expression and processing of APP in human BMECs.** (**A**) and (**B**) Senescence impairs α-processing of APP and enhances β-processing of APP. BACE1 inhibitor IV reverses the impairment caused by senescence by inhibiting Aβ generation and increasing expression of ADAM10 and sAPPα production. (**C**) and (**D**) Ang II impairs α-processing of APP in senescent cells. BACE1 inhibitor IV reverses reduced ADAM10 expression caused by Ang II, but does not affect sAPPα production, most likely as a result of decreased expression of APP. [↑= up-regulation, ↓=down-regulation, (-)=no effect]

First, treatment of young human BMECs with increasing concentrations (0.5nM and 1nM) of Ang II for 24h did not change protein levels of APP and ADAM10 (Supplemental Figure 2A-B). While Ang II caused an increase in expression of BACE1 (Supplemental Figure 2C), levels of Aβ40 were unchanged (Supplemental Figure 2D, Aβ42 production was undetectable, n=11, data not shown). Inhibition of angiotensin II type 2 receptor (AT_2_R) by PD123319 (but not AT_1_R inhibition by losartan), blocked the stimulatory effect of Ang II on expression of BACE1 (Supplemental Figure 2E). Moreover, treatment with nuclear factor κB (NF-κB) inhibitor, PDTC, rather than protein kinase C (PKC) inhibitor, Ro-31-8220, reversed Ang II-induced enhancement of BACE1 protein expression (Supplemental Figure 2F). We next determined the effects of Ang II on senescent human BMECs. Interestingly, ADAM10 protein levels were significantly reduced (Figure 4A). This effect was abolished by AT_2_R antagonist PD123319 (but not AT_1_R antagonist losartan; Figure 4B). More importantly, production of sAPPα was statistically lower following Ang II treatment (Figure 4C). No changes in APP or BACE1 expression were seen in senescent cells treated with Ang II (Figure 4D-E). Since BACE1 inhibitor IV increased ADAM10 expression and sAPPα production in senescent cells, we tested the possibility that BACE1 inhibitor IV reverses the impaired expression of ADAM10 and production of sAPPα caused by Ang II treatment. Our data showed that BACE1 inhibitor IV significantly elevated ADAM10 expression in cells treated with Ang II (Figure 5A), however it did not recover reduced sAPPα caused by Ang II (Figure 5B). Interestingly, the protein levels of APP were significantly decreased in cells treated with Ang II plus BACE1 inhibitor IV (Figure 5C). Schematic summaries of effects of Ang II or Ang II combined with BACE1 inhibitor IV on α-processing of APP in senescent cells are shown in Figure 6C and Figure 6D.

## DISCUSSION

In the present study, we report several novel findings. First, senescence impairs the expression of APP in human BMECs. Although cellular senescence does not decrease α-secretase expression, the production of sAPPα is significantly reduced, most likely as a result of decreased expression of APP protein. Second, BACE1 protein levels are elevated in senescent human BMECs. Consistent with increased BACE1, production of Aβ40 is significantly up-regulated. Third, both in young and senescent human BMECs, treatment with BACE1 inhibitor IV reduces Aβ generation and increases ADAM10 levels. However, production of sAPPα is only elevated in senescent BMECs. Fourth, AT_2_R/NF-κB signaling is involved in Ang II-induced enhancement of BACE1 expression in young endothelial cells. In contrast, Ang II suppresses ADAM10 protein levels by activation of AT_2_R thereby decreasing sAPPα production in senescent cells. These observations suggest that in endothelium AT_2_R is responsible for the effects of Ang II on APP metabolism. Senescent human BMECs are more sensitive to inhibitory effect of Ang II on α-processing of APP, and that loss of sAPPα caused by Ang II may impair the function of senescent human cerebrovascular endothelium. Finally, BACE1 inhibitor IV reverses decreased ADAM10 expression caused by Ang II, thereby restoring normal expression of ADAM10.

Vascular aging is characterized by endothelial dysfunction, thickened intima-media layer, enlarged vascular lumen, increased vascular stiffness, predisposition to development of atherosclerosis, decreased angiogenesis and aberrant response to stimuli [43,44]. Senescent endothelial cells have several major functional abnormalities, such as loss of replicative potential and increased susceptibility to injury [43]. Although the exact molecular mechanisms linking endothelial senescence with vascular aging are poorly understood, existing evidence suggests that senescent endothelial cells significantly contribute to age-related vascular disease [43,45,46].

Previous studies have demonstrated that APP is highly expressed in vascular endothelium and that intraluminal release of the APP cleavage products may play an important role in vascular homeostasis [14,16,47]. In the present study, passaging of cultured human BMECs resulted in senescence, including slower growth rates and increased expression of p16^INK4a^ and SA-β-Gal activity. Most notably, our studies are the first to demonstrate that in senescent human brain microvascular endothelium, the progressive decrease in sAPPα is associated with a reduced expression of APP. While we do not have an exact explanation for the decreased APP levels, it was shown that age-associated decreased maturation of APP in human fibroblasts was directly affected by increased level of intracellular cholesterol [13]. Of note, the expression of ADAM10 is unchanged and ADAM9 is increased in senescent endothelium. The mechanisms of APP processing by ADAM9 are not fully understood [42,48]. Controversial observations have been reported, demonstrating that there is an association between ADAM9 and activity of ADAM10 [49–51], however, the exact effect of increased expression of ADAM9 on ADAM10 protease activity in senescent endothelial cells remains to be determined.

In neuronal tissue, inadequate sAPPα levels may be sufficient to polarize APP processing toward the amyloidogenic pathway [52,53]. Consistent with this concept, in senescent human BMECs, loss of sAPPα is associated with significant increase in BACE1 expression and Aβ40 levels. Existing evidence indicates that deposition of Aβ exerts detrimental effects on cerebral blood vessels and impairs endothelial structure and function [54–56]. Since BACE1 is the rate limiting enzyme in the production of Aβ, BACE1 inhibitors have emerged as an attractive therapeutic approach in prevention and treatment of AD [21,57,58]. Because α- and β-secretase appear to compete for the intracellular pool of APP, sAPPα is increased upon BACE1 inhibition in neuronal cells [20,59]. In our studies, as expected, BACE1 inhibitor IV significantly decreased the generation of Aβ40 both in young and senescent human BMECs. However, we wish to emphasize that our findings are the first to demonstrate that BACE1 inhibitor IV increased sAPPα levels by elevating ADAM10 expression in senescent human brain microvascular endothelium. Currently, we do not have an explanation as to why increased expression of ADAM10 did not result in increased production of sAPPα in young endothelial cells in response to BACE1 inhibitor IV. Nevertheless, our findings suggest that BACE1 inhibitors may exert beneficial effects in senescent human BMECs by shifting APP processing to α-pathway. Further studies will be needed to determine the exact mechanism responsible for elevated ADAM10 expression caused by BACE1 inhibitors.

In the present study, we also examined the effects of Ang II on expression and processing of APP in young and senescent human BMECs. Based on the circulating levels of Ang II detected in patients with hypertension, 0.5nM and 1nM concentrations of Ang II were used [60,61]. Both AT_1_R and AT_2_R protein are expressed in cultured human cerebral microvascular endothelium [62,63]. AT_1_R has been shown to mediate majority of the physiological and pathological actions of Ang II [64]. The effects mediated by activation of AT_2_R in endothelial cells are not completely understood [65,66]. Interestingly, our findings demonstrate that in young human BMECs Ang II increased the expression of BACE1 by activation of AT_2_R. Existing evidence suggests that transcription factor NF-κB plays a role in regulation of BACE1 protein expression [67]. Furthermore, in rat glomerular endothelial cells Ang II activates NF-κB by AT_2_R-dependent signaling [68]. Of note, existing literature suggests that activation of PKC could also up-regulate BACE1 protein levels in endothelial cells [69]. In our experiments, treatment with NF-κB inhibitor, PDTC, (but not PKC inhibitor, Ro-31-8220), attenuated Ang II-induced up-regulation of BACE1 expression. This observation implies that NF-κB signaling is involved in Ang II-induced enhancement of BACE1 expression. Although the expression of BACE1 was increased, we still did not observe any changes in production and release of Aβ peptides. The exact mechanisms responsible for the discrepancy between increased expression of BACE1 and unchanged Aβ levels are currently unknown and remain to be determined. Interestingly, in senescent human BMECs, Ang II did not increase expression of BACE1. It is possible that because the levels of BACE1 protein have already been elevated in senescent human BMECs, treatment with Ang II could not further increase expression of BACE1. However, Ang II decreased ADAM10 protein levels in senescent endothelium. This effect was also dependent on activation of AT_2_R. Besides decreased ADAM10, Ang II also significantly reduced sAPPα generation in senescent endothelium. It is important to note that no previous study has characterized the effects of sAPPα on cerebrovascular endothelium. However, recent studies suggest that in neuronal cells, sAPPα exerts inhibitory effect on β-processing of APP [52,53]. Therefore, reduced sAPPα production by endothelium might impair the balance between non-amyloidogenic and amyloidogenic processing of APP. The impairment of α-processing and potential shift to β-processing of APP may help to explain dysfunction of senescent endothelium in response to Ang II. Moreover, endothelium-derived sAPPα contains KPI domain that has anticoagulation function responsible for protection against thrombosis in cerebral circulation [14,70]. More importantly, our recent studies have demonstrated that sAPPα production in cerebral endothelial cells affects levels of sAPPα in the hippocampus [16,71]. Since sAPPα is a well-known neuroprotective and neurotrophic molecule [14], cerebral endothelium-derived sAPPα may significantly contribute to protection of hippocampal function. Therefore our findings indicate that in the senescent endothelium loss of sAPPα induced by Ang II may increase vulnerability of vascular and neuronal cells to injury. Finally, we wish to point out that in order to prevent decreased ADAM10 expression and sAPPα levels caused by Ang II, senescent endothelial cells were treated with a combination of Ang II and BACE1 inhibitor IV. We observed a significant increase in ADAM10 expression during combined treatment. However, the levels of sAPPα were not recovered, most likely as a result of decreased expression of APP.

Taken together, the results of the present study suggest that reduced APP expression contributes to down-regulation of sAPPα in senescent brain microvascular endothelium. Increased BACE1 expression and Aβ production suggest that senescence promotes β-processing of APP. Treatment with BACE1 inhibitor IV is beneficial for senescent human BMECs. This effect is mediated by shifting of APP processing towards non-amyloidogenic pathway. The present study also reports a novel observation regarding the detrimental effects of Ang II on α-processing of APP by activation of AT_2_R in senescent human BMECs. Given the fact that the cleavage products of APP play an important role in vascular homeostasis, we propose that increased Aβ production together with loss of sAPPα are previously unrecognized mechanisms of cerebral microvascular endothelial dysfunction induced by senescence and Ang II. Our findings support the concept that pathological expression and processing of APP in senescent cerebrovascular endothelium may play an important role in pathogenesis of cerebral amyloid angiopathy and AD.

## METHODS

### Cell culture

Primary human BMECs were purchased from Applied Cell Biology Research Institute (Kirkland, WA). Human BMECs were grown in endothelial growth medium 2 (EGM2; Lonza, Allendale, NJ) which contained: endothelial basal medium 2 (EBM2; Lonza, Allendale, NJ) supplemented with 2% fetal bovine serum, fibroblast growth factor, vascular endothelial growth factor, insulin-like growth factor, epidermal growth factor, ascorbic acid, hydrocortisone, bac off and heparin.

Senescence of cells was induced by serial passages [13,72]. Cells were regarded as young at passage 5 (P5) and senescent at passage 15 (P15) [72]. We generated growth curves using P5 and P15 human BMECs by counting cell number every 24h for 5 days. At day 0, we plated 2.5x10^4^ human BMECs per 100mm dish and changed media every day [73].

In some experiments, human BMECs were treated with or without increasing concentrations of BACE1 inhibitor IV (0.1μM, 0.5μM, 5μM) (Calbiochem, Billerica, MA) [57] or human Ang II (0.5nM, 1nM) (Sigma Aldrich, St. Louis, MO) [60,61] for 24h. For Ang II receptor/pathway blockade, human BMECs were treated with AT_1_R antagonist, losartan (1μM) (Cayman, Ann Arbor, MI) [68], or AT_2_R antagonist, PD123319 (1μM) (Cayman, Ann Arbor, MI) [68], or NF-κB inhibitor, PDTC (50μM) (Calbiochem, Billerica, MA) [74], or PKC inhibitor, Ro-31-8220 (1μM) (Calbiochem, Billerica, MA) [69,75] for 1h prior to Ang II treatment.

### Senescence-associated β-Galactosidase staining (SA-β-Gal)

We stained P5 and P15 human BMECs for SA-β-Gal activity according to manufacturer’s protocol (BioVision, Milpitas, CA). The percentage of senescent cells was calculated by the total number of senescent cells (blue color) divided by the total number of cells counted under a microscope (Nikon Eclipse TE2000-U Microscope, 150x total magnification) [73].

### Western blot analysis

To perform Western blot analysis, cells were collected and lysed in ice cold lysis buffer [10mM HEPES, 50mM NaF, 50mM NaCl, 5mM EDTA, 5mM EGTA, 100μM Na_3_VO_4_, 50mM Na pyrophosphate, 1% Triton X-100, pH 7.4, and protease inhibitor cocktail (Sigma, St. Louis, MO)] as previously described [16]. Equal protein amounts were resolved by SDS-PAGE and transferred to nitrocellulose membranes. Blots were probed with APP (Invitrogen, Carlsbad, CA), BACE1 (Abcam, Cambridge, MA), ADAM10, ADAM17 (Millipore, Billerica, MA), ADAM9 (Cell Signaling, Danvers, MA), p16^INK4a^ (Cell Applications, San Diego, CA) and β-actin (Sigma Aldrich, St. Louis, MO) specific primary antibodies. Protein expression was normalized to β-actin. For α-secretase, the protein levels of mature ADAM10, mature ADAM9, and mature ADAM17 (lower bands on the blots) were quantified.

### ELISA for sAPPα, Aβ40, and Aβ42

Secreted sAPPα, Aβ40, and Aβ42 from cell supernatant were measured using human sAPPα high sensitive assay kit (Immuno-Biological Laboratories-America, Minneapolis, MN), human Aβ40 ELISA kit, and human Aβ42 ELISA kit (Invitrogen, Camarillo, CA), respectively, following manufacturer’s protocols.

### Statistical analysis

Data are presented as mean±SEM. Differences between mean values of multiple groups were analyzed using one-way ANOVA followed by Tukey test (SigmaStat 12.0 for Windows). The growth curves of different passages were compared on individual days. Unpaired Student *t*-test was used to analyze comparison between two groups. P<0.05 was considered statistically significant.

## Supplementary Material

Supplementary File
