# Supplementary material for: Effects of senescence and angiotensin II on expression and processing of amyloid precursor protein in human cerebral microvascular endothelial cells
Source: Aging (Albany NY). 2018 Jan 15;10(1):100–14. doi: 10.18632/aging.101362 (PMC5811245; doi:10.18632/aging.101362)
Supplement: Supplementary File [file aging-10-101362-s001.pdf]

SUPPLEMENTARY MATERIAL

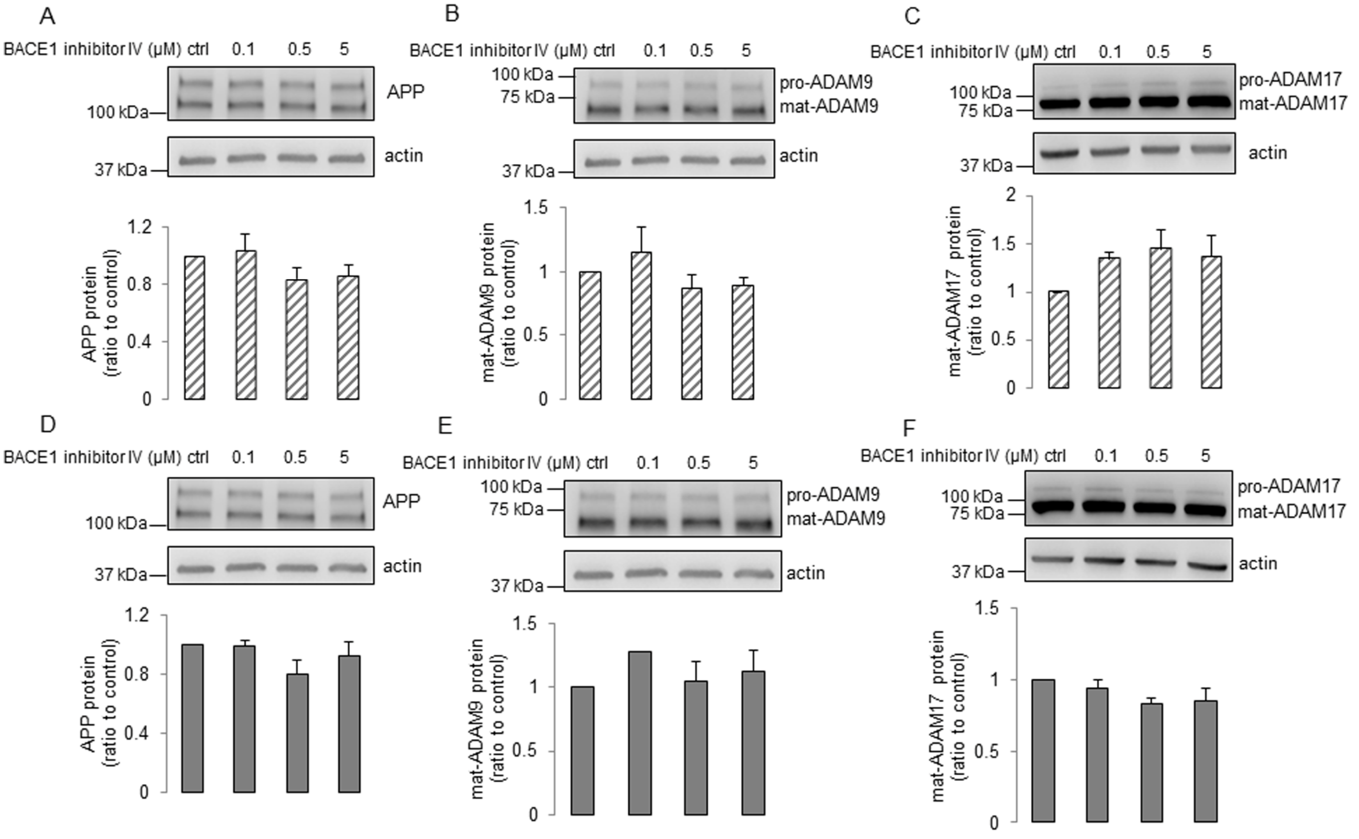

Supplementary Figure 1. APP, ADAM9 and ADAM17 protein levels were unchanged by BACE1 inhibitor IV treatment of young and senescent human BMECs. P5 cells (**A**, **B** and **C**) and P15 cells (**D**, **E** and **F**) were treated with BACE1 inhibitor IV with indicated concentrations for 24h. Cell lysates were subjected to Western blot. Protein levels of APP (**A** and **D**, n=5), ADAM9 (**B** and **E**, n=3) and ADAM17 (**C** and **F**, n=3) were measured. Data are presented as mean±SEM.

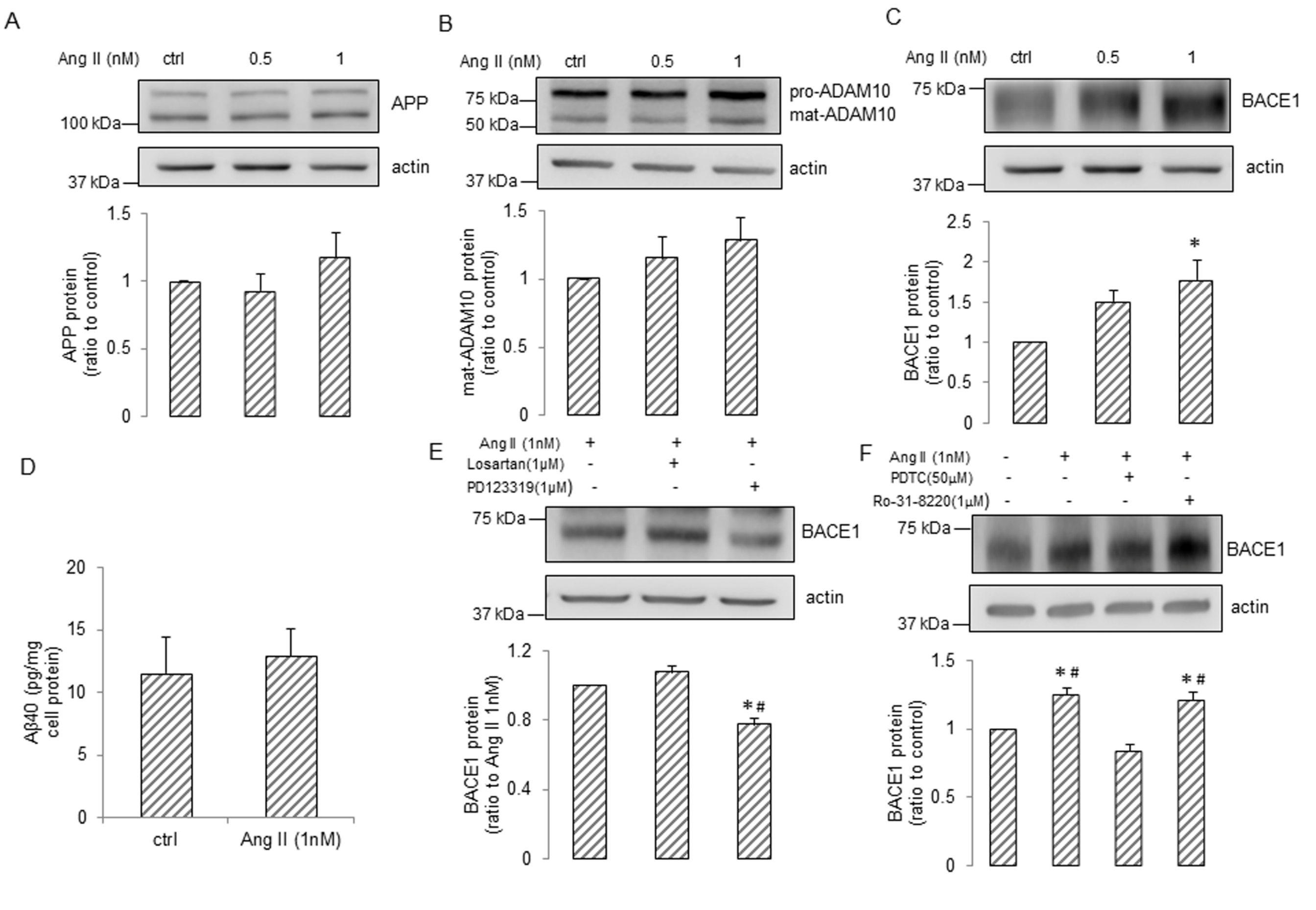

Supplementary Figure 2. The effects of Ang II on expression and processing of APP in young human BMECs. P5 cells were treated with Ang II with indicated concentrations for 24h. Cell lysates were subjected to Western blot. Protein levels of (**A**) APP, n=7-9, (**B**) ADAM10, n=7-9 and (**C**) BACE1, n=9-14 were measured. *P<0.05, compared to control. (**D**) BMECs were cultured in the absence or presence of 1nM Ang II for 24h. Conditioned media (2ml) were collect for measuring Aβ40 via a commercially available ELISA kit, n=11. (**E**) Cells were treated with losartan (10^{-6}M) or PD123319 (10^{-6}M) for 1h, and then incubated with Ang II (1nM) for 24h. BACE1 expression was measured, n=7. *P<0.05, compared to Ang II (1nM), $^{\#}$P<0.05, compared to Ang II (1nM) plus losartan (10^{-6}M). (**F**) Cells were treated with PDTC (50μM) or Ro-31-8220 (1μM) for 1h, and then incubated with Ang II (1nM) for 24h. BACE1 expression was measured, n=6. *P<0.05, compared to control, $^{\#}$P<0.05, compared to Ang II (1nM) plus PDTC (50μM). Data are presented as mean±SEM.
